# Synthetic approaches to metal-coordination-directed macrocyclic complexes

**DOI:** 10.3389/fchem.2022.1078432

**Published:** 2022-11-24

**Authors:** Qingqing Fang, Yan Xu, Xiaosheng Yan, Tao Jiang, Yunbao Jiang

**Affiliations:** ^1^ Department of Chemistry, College of Chemistry and Chemical Engineering, The MOE Key Laboratory of Spectrochemical Analysis and Instrumentation, and iChEM, Xiamen University, Xiamen, China; ^2^ Songshan Academy, Zhengzhou University of Aeronautics, Zhengzhou, China; ^3^ School of Pharmaceutical Sciences, Xiamen University, Xiamen, China

**Keywords:** macrocycles, metal coordination, synthesis, foldable ligands, amphiphiles

## Abstract

Metal-coordination-directed macrocyclic complexes, in which macrocyclic architectures are formed by metal-ligand coordination interactions, have emerged as attractive supramolecular scaffolds for the creation of materials for applications in biosensing and therapeutics. Despite recent progress, uncontrolled multicyclic cages and linear oligomers/polymers is the most likely outcome from metal-ligands assembly, representing a challenge to current synthetic methods. Herein we outlined the state-of-art synthetic approaches to the metal-coordination-directed macrocyclic complexes by using foldable ligands or through assembly of amphiphilic ligands. This mini-review offers a guideline for the efficient preparation of metal-coordination-directed macrocyclic complexes with predictable and controllable structures, which may find applications in many biology-related areas.

## Introduction

Macrocycles with metal ions are of particular interest in biological and materials sciences. Natural occurring metal-containing macrocycles are essential to the regulation of key cations in many living organisms and the feasibility of using labile metal coordination interactions to form complex architectures. From the perspective of synthetic materials, the directed cyclization enables the creation of various complex structures using simple molecular building blocks. The metal-coordinated macrocycles can be classified into two types: 1) the macrocycles with metal ions chelated at the central cavities; 2) the assembled macrocycles from which metal ions serve as the linkers to connect multiple ligand structures into cyclic architectures (Thakur et al., 2022). The first type of macrocycles is ubiquitous in nature, such as cytochrome C, chlorophyll, vitamin B_12_ (Lindoy et al., 2013; Joshi et al., 2015). A variety of their synthetic analogues has also been synthesized with considerable success, using macrocycles with inward metal ion coordination sites (Edwards et al., 2006; Kubota et al., 2016; Feng et al., 2017; Yogendra et al., 2017; Haaf et al., 2021; Wang et al., 2021; Bodman et al., 2022; Muravev et al., 2022; Nystrom et al., 2022; Wang et al., 2022). By contrast, the synthesis of the second type of macrocycles remains a challenging task. This is because that most ligand precursors preferred extended conformation, generating significant entropy penalty during the cyclization process (White and Yudin, 2011; Chow et al., 2019; Mortensen et al., 2019). The resultant syntheses usually led to linear oligomers/polymers or uncontrolled multicyclic cages as the major byproducts, in addition to the prescribed cyclic molecular structures.

Despite the synthetic difficulty, metal-coordination-directed macrocyclic complexes have attracted the attentions of supramolecular chemists, owing to their pre-organized cyclic binding cavity where multiple binding sites enhance binding affinity and selectivity for a guest species (Cabbiness and Margerum, 1969; Taylor et al., 2012; Marti-Centelles et al., 2015). In this mini-review, we highlighted modern synthetic approaches to metal-coordination-directed macrocyclic complexes with improved yields, using foldable and amphiphilic ligands, respectively.

### Cyclization using foldable ligands

**FIGURE 1:**
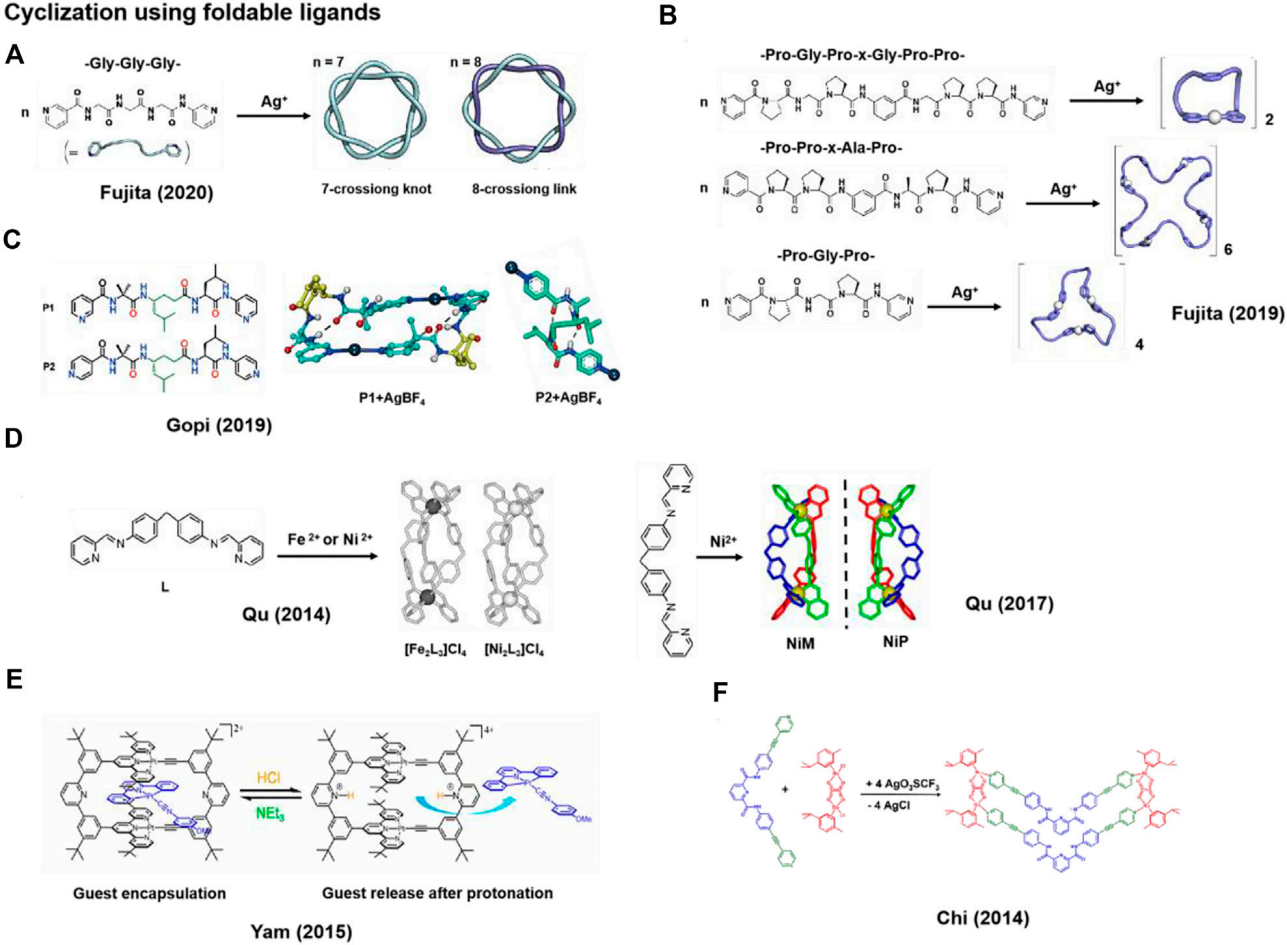
Representative metal-coordinated macrocycles synthesized using foldable ligands. **(A,B)** Macrocyclization via coordination between Ag^+^ and foldable peptides; **(C)** Structures of folded peptides **P1** and **P2**, and the crystal structures of **P1**+Ag^+^ (2 + 2 macrocycle) and **P2**+Ag^+^ complexes; **(D–F)** Structures of ligands containing aromatic ring and pyridine groups for the coordination with metal ions to form macrocycles; **(D)** Structures of ligand **L**, *M*- and *P*-enantiomer coordinated with Fe^2+^ or Ni^2+^ to form **[Fe**_**2**_ **L**_**3**_ **]Cl**_**4**_, **[Ni**_**2**_ **L**_**3**_ **]Cl**_**4**_, **NiM** and **NiP**; **(E)** pH-responsive Pt^2+^ directed macrocycles; **(F)** The formation of macrocyclic octahedral Ru(II) complexes. Adapted with permission from refs (Li et al., 2014; Mishra et al., 2014; Chan et al., 2015; Qin et al., 2017; Sawada et al., 2019a; Misra et al., 2019; Inomata et al., 2020).

The conformation of ligands dictates the outcome of metal-coordination driven self-assembly. The formation of macrocyclic metal complexes typically requires the ligand to exist in a folded conformation. This can be achieved by using flexible yet conformation-tunable ligands. Fujita and co-workers employed flexible but foldable short peptide as ligands with appended metal-coordination sites, *i.e.*, pyridine motifs, at both the C- and N-termini. Upon Ag^+^ coordination, 7-/8-crossion knot or helical complexes can be generated, which further self-assemble into larger structures (Sawada et al., 2019a; Sawada et al., 2019b; Inomata et al., 2020; Inomata et al., 2021) (Figures 1A,B). However, attempts to generate similar topologies using longer peptide chains were unsuccessful because of the unfavorable thermodynamics and kinetics. The results also demonstrated that the reversible function group modified in flexible short peptide fragments would give rise to a new paradigm in metal-coordination driven self-assembly, facilitated by the entangling nature of peptides. Intriguingly, resembling the dynamic structural switching observed in biological systems, they also found that the structure of metal-coordination-directed macrocyclic complexes could be changed from catenane structure to twisted macrocycle by replacing the metal ion trapped at the center of macrocyclic structure (Sawada et al., 2017).

The formation of metal-coordinated-directed complexes can be regulated by coordination site of the ligand (Fisher and Gellman, 2016; Lee et al., 2016). Gopi et al. designed α,γ-hybrid peptides **P1** and **P2** where the termini were modified with 3- or 4-pyridinyl groups for metal coordination (Figure 1C) and the N-terminal 2-aminoisobutyric acid (Aib) residue facilitates the helical conformation (Misra et al., 2019). Both the **P1** and **P2** adopt a right-handed 12-helix structure maintained by two consecutive intramolecular 12-membered ring hydrogen bonds. Upon the coordination with Ag^+^, **P1** forms a 2 + 2 macrocycle, and the right-handed 12-helix structure transforms into a left-handed helix with only one intramolecular 12-membered ring hydrogen bond. However, **P2** forms a porous metal-helix framework in the presence of Ag^+^, in which the original 12-helix structure remains. Despite the same sequence of **P1** and **P2**, the difference in the assembly outcome demonstrates the important role of coordination site in directing the generation of metal-coordination-directed macrocyclic complexes.

Ligands containing rigid aromatic units and modified with pyridine, which feature a propensity of bending, can easily coordinate with metal ions to generate macrocyclic complexes. Based on the pyridine-containing ligands, Qu et al. (Yu et al., 2012; Li et al., 2014) and Yam et al. (Chan et al., 2015) (Figures 1D,E) obtained several Pt, Fe, Ni ions coordinated macrocyclic complexes through the interactions between nitrogen atom on the ligands and metal ions. Notably, the rigid structure of aromatic ring in the backbone allows for guest binding via π-π and metal-metal interactions.

Qu and co-workers reported two metal-coordinated macrocycles **[Ni**_**2**_ **L**_**3**_ **]Cl**_**4**_ and **[Fe**_**2**_ **L**_**3**_ **]Cl**_**4**_ that consist of similar bimetallic triple helical structures (Figure 1D) (Li et al., 2014). The accumulation of extracellular amyloid β-peptide (Aβ) is a pathological hallmark of Alzheimer’s disease (AD) (Barbour et al., 2021; Ottoy et al., 2021). Recently, some studies proposed that the binding of heme b and Aβ to generate Aβ-complex maybe correlate with the onset of AD (Schipper et al., 1995; Atamna and Frey, 2007; Wang et al., 2013). It is worth noting that **[Ni**_**2**_ **L**_**3**_ **] Cl**_**4**_ and **[Fe**_**2**_ **L**_**3**_ **] Cl**_**4**_ could target 13–23 α/β-discordant region of the Aβ peptide for inhibiting Aβ aggregation. Furthermore, both the complexes showed higher binding affinities to Aβ than heme. This means that it can act as inhibitors to affect heme excessive synthesis, which was induced by Aβ and reduced the peroxidase activity of Aβ-heme. For PC12 cell experiments, these metal complexes exhibited little toxicity and suppressed the excess biosynthesis of heme. Therefore, the metal-coordination-directed macrocyclic complexes can not only function as potential agents against AD, but also provide new insights into the biological effects of Aβ inhibitors.

Intriguingly, based on the same approach that utilizes angled aromatic structure of pyridine groups as ligands, Qu and co-workers also developed chiral metal-coordination-directed macrocyclic complexes **NiP** that can inhibit the growth of breast cancer stem cells (CSCs) specifically and have little side effects on normal cells, whereas its enantiomer **NiM** is inactive (Figure 1D) (Qin et al., 2017). Telomeres play an important role in cell survival, and its damage and shortening will cause cell apoptosis (Sfeir and De Lange, 2012). In this work, the steric configuration of **NiP** exhibit higher selectivity toward telomere G-quadruplex DNA (G4-DNA) in breast CSCs than **NiM**. Besides, the cationic Ni^2+^ centers in the metal-coordination complexes can bind to telomere G4-DNA *via* electrostatic interaction. The preferential interaction between **NiP** and G4-DNA resulted in the dissociation of telomere associated protein from telomeres, the damaging of telomere DNA and cancer cells apoptosis. The study demonstrates that the ability of chiral metal-coordination-directed macrocyclic complexes to function as chiral drugs for tumor treatment.

The excellent guest selectivity of metal-coordination-directed macrocyclic complexes facilitates application in drug delivery. Yam and co-workers designed a series of cyclic rectangles of Pt^2+^-terpyridine to explore the binding and release of planar Pt^2+^, Pd^2+^, Au^3+^ and Au^+^ complexes *via* metal-metal, π-π and electrostatic interactions. These complexes serve as anticancer drugs (Figure 1E) (Chan et al., 2015). In this work, bis-alkynyl ligands containing several aromatic rings of bended conformation cap the U-shaped di-Pt^2+^ terpyridine moiety at the end. The folded conformation of ligands promotes the formation of rigid plane and ring structures. In comparison to the linear structure, tuning ring size of the metal-coordinated-directed complexes also provides the possibility of exploring host-guest chemistry. In addition, the pH-responsive functional group pyridine was introduced into cyclic rectangle. With a change of the pH, protonation or deprotonation of the pyridine nitrogen atoms would trigger guest release and capture reversibly, operating in a lock and key mechanism. The size of the ring backbone and the presence of functionality groups can be easily modulated and modified, which can expand the application of metal-coordination-directed macrocyclic complexes in the area of controllable delivery of drugs.

Moreover, Chi et al. used ligands containing aromatic units such as dipyridyl to construct macrocyclic arene-Ru complexes. The central scaffolds of the dipyridyl ligand display a 120° geometry upon its coordination with Ru metal ion on the two ligand moieties. The self-assembly between dipyridyl ligand and diruthenium arene complex furnishes a [2 + 2] metallacycle structure that is reminiscent of a wedge. Encouragingly, the ditopic ligand of this macrocyclic arene-Ru complexes can interact with enhanced green fluorescent protein (EGFP) (Figure 1F) (Mishra et al., 2014). The photophysical properties of green fluorescent protein (GFP) are sensitive to the change of protein conformations. The central pyridy-2,6-dicarboxamide moiety of ligand can interact with amino acid residues Arg168 of EGFP through hydrogen bond, leading to a conformational change in EGFP and the resultant emission quench, while no significant change were found for other proteins. Moreover, for cell experiments, the fluorescent emission of GFP was completely quenched after bacterial cells were incubated with the macrocyclic arene-Ru complexes after 6 h. This work not only expands the design of sensors for biomolecules but also affords a pathway to explore the mechanism of targeting protein drug delivery.

The synthetic approach of foldable or folded conformation of ligands is favored to guide the construction of various kinds of metal-coordinated-directed complexes. The conformation of ligands is significant to the cyclization, such as ligands with flexible and fine-tuning structure, the difference of coordination sites on ligands, as well as the bended structure of aromatic ligands that modified with pyridines. This approach imposes strict requirement on the structure of ligand, that the foldable conformation of ligands should feature a certain angularity to promote cyclization. However, for linear ligands, they would tend to form linear oligomers/polymers in the presence of metal ions. It means that we should develop the other approaches to favor the formation of metal-coordination-directed macrocyclic complexes between metal ions and flexible ligands.

### Cyclization using amphiphilic ligands

Inspired by self-assembly of amphiphilic surfactants forming micelles and vesicles, supramolecular amphiphiles formed by metal-coordination interactions have been developed to afford a novel approach to build metal-coordination-directed macrocyclic complexes, wherein the ligands can be flexible.

**FIGURE 2:**
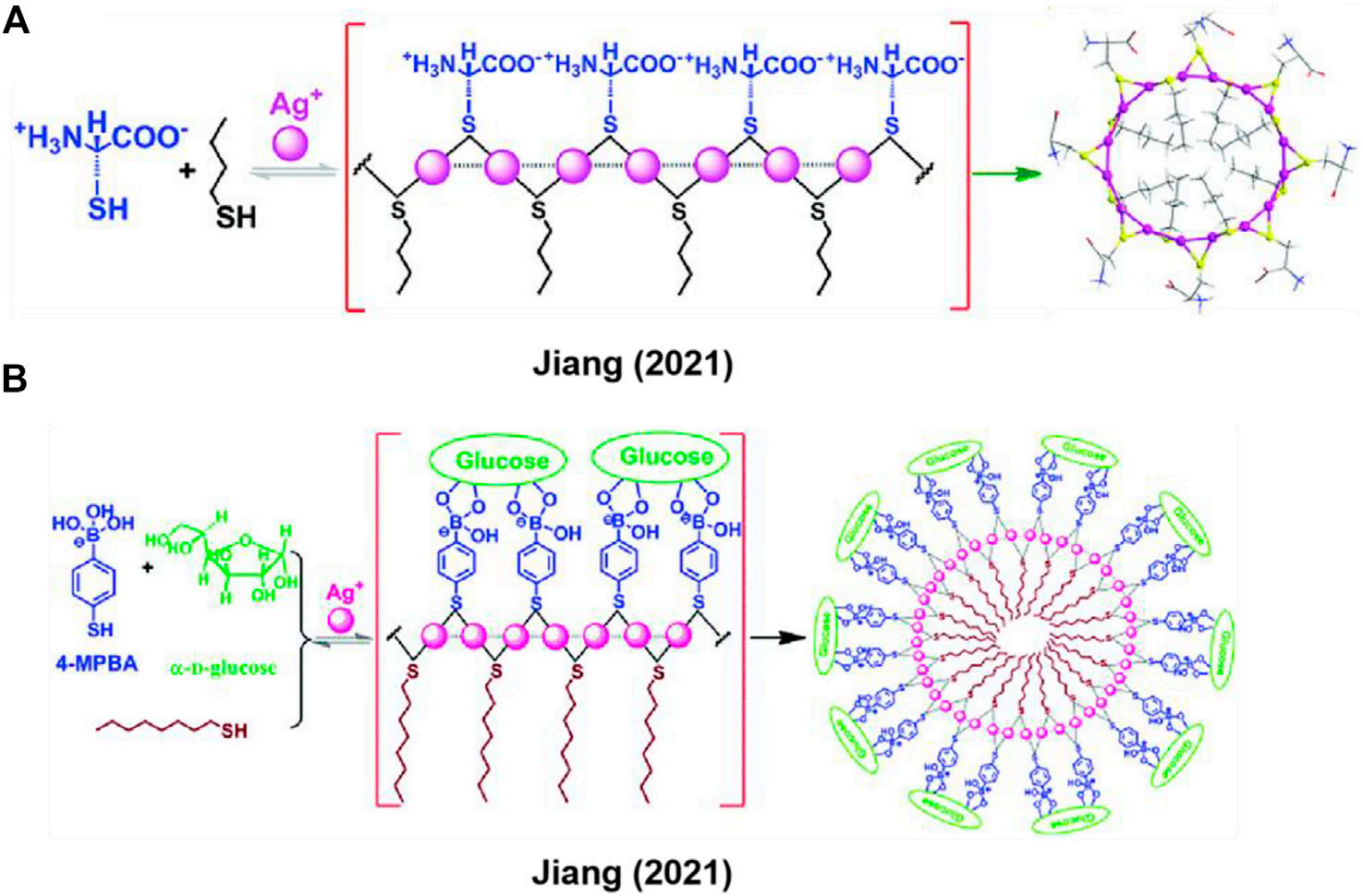
**(A,B)** Thiol-containing amphiphilic ligands coordinated with Ag^+^ to generate macrocyclic complexes. Adapted with permission from ref (Xu et al., 2021a; Xu et al., 2021b).

Previously, Jiang group has developed a series of *in-situ* formed Ag^+^-thiol linear coordination polymers by employing the cysteine derivatives as thiol ligands (Shen et al., 2009; Li et al., 2011; Shen et al., 2011; Zhang et al., 2015; Tao et al., 2017; Yuan et al., 2019). In addition to the coordination interaction of Ag^+^ with thiolate ligand, argentophilic interaction (Ag^+^···Ag^+^) and electrostatic interaction, π-π interactions between side-chains of ligands could also facilitate the formation of the coordination polymers. Recently, Jiang et al. (Xu et al., 2021a) utilized hydrophilic and hydrophobic thiol ligands to construct metal-coordination-directed macrocyclic complexes (Figure 2A). In this work, Ag^+^, hydrophilic cysteine (Cys) and hydrophobic *n*-butylmercaptan (*n*-BuSH) formed Ag^+^-thiol coordination polymers, the absorption band at 285 nm demonstrated that Ag^+^ on the backbone linked by localized Ag^+^···Ag^+^ interaction. Cys and *n*-BuSH served as building blocks located on the outward and inward of complexes backbone, respectively. By virtue of the hydrophilic-hydrophobic interactions and Ag^+^···Ag^+^ interaction, the contrivable ligand as block unit and metal ion directed the formation of macrocyclic complexes. For most of supramolecular polymers, they display majority rules. Intriguingly, in this work, the chirality of Cys ligand is transferred to the macrocyclic backbone, and the metal-coordination-directed complexes exhibit an unusual anti-Z-shaped CD-*ee* dependence, previously termed as a “racemate rules effect” (Wu et al., 2014; Chen et al., 2016; Yan et al., 2020; Xu et al., 2021a), opposite to the conventional *Z*- or S-shaped CD-*ee* curve that governed by the majority rules (Green et al., 1995; Smulders et al., 2010; Cao et al., 2013). This leads to high accuracy in chiroptically measuring *ee* of chiral analytes at high value ends, and provides a new strategy for accurate and simple determination of *ee* of asymmetric synthesis products (Chen et al., 2016).

Based on the strategy of co-assembling hydrophilic and hydrophobic ligands into the Ag^+^-thiol coordination polymer, Jiang et al. (Xu et al., 2021b) employed the hydrophilic 4-mercantophenyboronic acid (4-MPBA) and hydrophobic 1-octanethiol to generate larger metal-coordination-directed macrocyclic complexes (Figure 2B), wherein glucose could be bound to two neighboring boronic acid groups form 4-MPBAs on the Ag^+^ coordination polymeric backbone. This binding event could facilitate the formation of the cyclic backbone *via* the achievement of a good hydrophilic-hydrophobic balance. By forming a CD-active assembly, the molecular chirality of glucose is expressed as supramolecular chirality. Moreover, dynamic light scattering (DLS) measurements demonstrated that the metal complexes formed in the presence of glucose are more monodisperse than those formed by other monosaccharides of similar structures. The synthetic strategy of supramolecular amphiphiles *in-situ* triggered by metal ions is applicable to constructing metal-coordination-directed macrocyclic complexes, which also can be used for recognizing biomolecules such as glucose.

Comparing with the complex synthetic strategies of macrocyclic compounds, *in-situ* formed metal-coordination-directed macrocyclic complexes are easy to obtain and show unique merits. However, it is of grand challenge to obtain macrocyclic complexes of precisely uniform size. There are also difficulties in structural characterizations as the complexes can conglomerate in drying process.

## Conclusion

We summarized two types of metal-coordination-directed macrocyclic complexes that have been synthesized efficiently based on smart ligand choices. 1) A variety of macrocycles were described using the carefully designed ligands with folded structures that geometrically satisfy desired ring topology. Many of the resultant macrocycles have found applications in biology-related areas, including biomolecular recognition, drug delivery, and therapeutics for AD and cancer. 2) Cyclic metal-ligand arrays generated by exploiting micellar and vesicular assembly of flexible amphiphilic ligands in the presence of metal ions.

There are significant opportunities and challenges in the area of metal-coordination-directed macrocyclic complexes. To achieve real-world applications such as clinical diagnosis and therapeutics, a key future research direction is to develop easily synthesized systems that can be derivatized with functional units such as biomolecule recognition sites and optical sensing units. In the design of macrocyclic metal complexes, the cooperative effect of multiple interactions, such as π-π interactions, metal-metal interactions and hydrogen bonding have been proved to benefit the construction of metal-coordination-directed macrocyclic complexes. In this context, other interactions, such as halogen and chalcogen bonds, are less exploited and could be alternative driving forces to promote the synthesis of metal-coordination-directed macrocyclic complexes. The excellent properties of metal-coordination-directed macrocyclic complexes, for example, flexible and easy tuning structure create a special microenvironment to bind guest molecules and many functional modification sites have drawn significant attentions, leading to applications in biological field, such as molecular recognition and therapeutics. We anticipate this mini-review will facilitate the synthesis of metal-coordination-directed macrocyclic complexes with diverse structures and functions.
